# The Effects and Types of Parental Involvement in School-Based Sport and Health Programs Still Represent a Knowledge Gap: A Systematic Review

**DOI:** 10.3390/ijerph191912859

**Published:** 2022-10-07

**Authors:** Klára Kovács, Karolina Eszter Kovács, Katinka Bacskai, Zsolt Békési, Ádám József Oláh, Gabriella Pusztai

**Affiliations:** 1 MTA-DE-Parent-Teacher Cooperation Research Group, Institute of Educational Sciences and Cultural Management, University of Debrecen, 4032 Debrecen, Hungary; kovacs.klara@arts.unideb.hu (K.K.); bacskai.katinka@gmail.com (K.B.); bekesi422@gmail.com (Z.B.); pusztai.gabriella@arts.unideb.hu (G.P.); 2 MTA-DE-Parent-Teacher Cooperation Research Group, Institute of Psychology, University of Debrecen, 4032 Debrecen, Hungary; 3 MTA-DE-Parent-Teacher Cooperation Research Group, Institute of Sports Science, University of Debrecen, 4032 Debrecen, Hungary; olah98adam@gmail.com

**Keywords:** parental involvement, school-based sport and health programs, systematic review

## Abstract

Background: Health-related behaviours and attitudes acquired in childhood significantly shape health behaviours in adulthood and play an important role in preventing children from becoming overweight. Interventions incorporating parental involvement can provide outstanding support in shaping a child’s health-related behaviour. However, parental involvement has not been investigated from the perspective of efficacy except for obesity. Therefore, this systematic review investigates school-based physical activity intervention programmes incorporating parental involvement. We aim to explore the impact of these programmes and the parental involvement they provide on behaviours that influence child health, which are essential for preventing children and adults from becoming overweight and promoting health-conscious lifestyles. Methods: This systematic literature review follows PRISMA guidelines. The EBSCO Discovery Service Search Engine was used for searching for literature. Papers included met the following inclusion criteria: (1) reported original, empirical research or systematic review published in a peer-reviewed journal; (2) primary or secondary school age (6–18 years) as the target population; (3) examined school-based sport or health prevention and intervention programs; (4) only healthy children and youth in the indicated age group; (5) school-based prevention or intervention program; (6) examines parental involvement; (7) in the English language, and (8) in disciplines of education, psychology, social work, sociology, social sciences and humanities. Results: An extremely limited number of interventions of sufficient quality address the role of parental involvement (*N* = 17). The forms of parental involvement show a huge variety, ranging from leaflets, home packs, sports organisations “forced” by the pandemic, parent meetings, programmes, courses, and school programmes with diverse children over several months (cooking together, gardening, playing sports together, etc.). Therefore, it is difficult to measure their effectiveness and impact. Conclusions: The impact of parental involvement on children’s health behaviour, especially physical activity and nutrition, as two of the most important factors in preventing them from becoming overweight, are unclear, and other correlations, e.g., academic achievement, are scarce.

## 1. Introduction

The prevalence of childhood obesity has increased dramatically in recent years worldwide, making it essential to have programmes that focus on physical activity and nutrition, targeting energy balance-related behaviours [1]. Health-related behaviours and attitudes acquired in childhood, such as physical activity and dietary habits, are important determinants of health behaviours in adulthood and play an important role in preventing individuals from becoming overweight in childhood, adolescence and adulthood. Regular physical activity has several positive effects: it reduces the prevalence of many diseases (e.g., cardiovascular disease, diabetes, cancer, osteoporosis, etc.), obesity, insulin resistance, high BMI and hypertension, and contributes to increased self-confidence and the development of a range of skills and abilities [2,3]. It improves several skills, self-confidence, persistence, maturity, and social competencies, increasing school participation and students’ educational and other performance, thus contributing to students’ school achievement [4,5,6]. Schools have a very important role in preventing adolescent overweight/obesity: secondary schools should focus their health promotion efforts on healthy eating, involving parents/households and taking gender differences into account [7,8]. This makes it important to analyse and develop interventions to create effective programmes to increase physical activity to prevent individuals from becoming overweight. However, in previous analyses and reviews of interventions, the authors have concluded that it is unclear which types and forms of intervention programmes are considered effective. However, in general, multi-component interventions, particularly among adolescents and those involving school, family and community, have the potential to make a significant difference in physical activity levels and should be promoted [3,9].

The role of parents is undoubted in shaping their children’s health behaviours through model following and direct involvement, and parental influence is essential in shaping children’s healthy body image. Parental comments on weight and body size are among the most consistent factors contributing to a child’s self-image [10]. School intervention programmes that involve parents in the intervention appear to be more successful [11]. However, the form and method are not clear as to what has a real positive impact on shaping children’s physical activity and health-conscious eating and health behaviour. This is due to the different study designs, study quality and outcome measures used and the failure to involve parents [12]. It is important to note that the exact type and extent of parental involvement vary considerably, with evidence from pilot studies suggesting that parental information alone is often not sufficient [13]. Other analyses draw attention to the fact that the added value of parental involvement is questionable due to its paucity, that there are few studies on the subject, that most of these report inconsistent results, and that the form and content of parental involvement are often unclear [12]. However, parental involvement goes beyond contributing to the development of a child’s health-conscious lifestyle. Participation in school sports programmes has been shown to contribute effectively and creatively to parental participation in school, e.g., in the case of disadvantaged groups such as low socioeconomic status (SES) African Americans [14]. In addition, joint participation in intervention programmes can, of course, not only influence the child’s health, attitudes and lifestyle in a positive way for life but can also contribute to the health awareness of parents. Thus, programmes that change the perspective of the child and the family together, using the broader family health environment, should consider the underlying capacities of parents and the importance of joint goals and activities [15,16]. 

This systematic review aims to examine school-based physical activity intervention programmes incorporating parental involvement. We aim to explore the impact of these programmes and the parental involvement they provide on children’s health behaviours, particularly physical activity and nutrition, two key behaviours for preventing them from becoming overweight, which is essential for preventing childhood and adult obesity and promoting health-conscious lifestyles. Our analysis is unique because it specifically examines the impact of school sports and physical activity-based programmes related to parental involvement. At the same time, there are numerous studies and systematic reviews on the impact of parental involvement on academic achievement and the impact of school sports programmes on physical activity, and the combination of these topics makes our study unique.

## 2. Materials and Methods

This systematic literature review follows the Preferred Reporting Items for Systematic Reviews and Meta-Analyses (PRISMA) guidelines [17]. 

### 2.1. Literature Search

**Figure 1:**
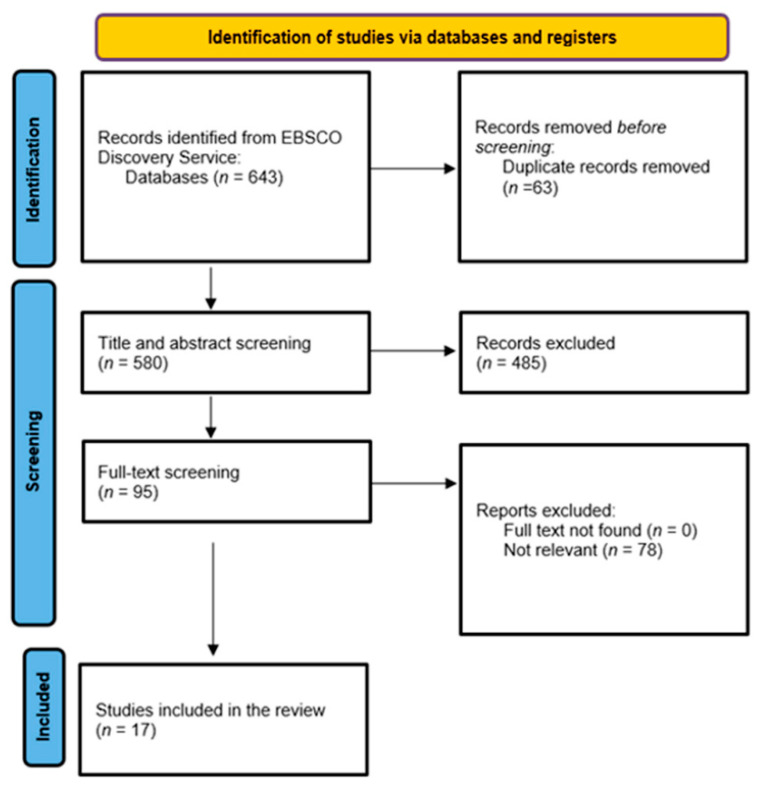
Preferred Reporting Items for Systematic Reviews and Meta-Analyses (PRISMA) diagram.

To find the optimal search strategy, we consulted a librarian at the University of Debrecen. The searches were performed on 16 November 2021 in the EBSCO Discovery Service Search Engine, which contains 85 databases. We used the following search strategy: parental involvement or parent engagement or parent participation AND “school-based sports” or “school sports” AND health AND physical activity or exercise AND “academic achievement” or “academic performance” or “academic engagement”. Our systematic searches resulted in a total of 643 records; after double filtering, 63 records were excluded. After removing duplicates, 580 studies were screened by their title and abstract. The full text of 95 records was assessed for eligibility based on the inclusion and exclusion criteria (see Section 2.2). Eventually, 17 studies focusing on the effects and types of parental involvement in school-based sport and health programs were included (see Figure 1).

### 2.2. Inclusion and Exclusion Criteria

Literature included in this systematic review met the following inclusion criteria: (1) reported original, empirical research or systematic review published in a peer-reviewed journal; (2) primary or secondary school age (approx. 6–18 years old) as target population; (3) examined school-based sport or health prevention and intervention programs (4) only healthy children and youth in the indicated age group; (5) must be school-based prevention or intervention program; (6) examines parental involvement; (7) in the English language, and (8) in the disciplines of education, psychology, social work, sociology, social sciences and humanities. In this study, we focused only on school-aged children (6 to 18 years); thus, other age groups were excluded. We examined only journal articles, while books, book chapters, dissertations and newspaper articles were excluded following international practice. Although parental involvement and parent-child interaction and relationships may be culturally different, the systematic review did not focus on specific countries or regions to provide the opportunity for finding culturally different perspectives of parental involvement programs. Only English papers were involved in the study according to international practice, which ensures that publications are scientifically sound and of an appropriate professional standard (see the examples of O’Connor et al. [2] and Verjans-Janssen et al. [11]).

### 2.3. Data Extraction and Assessment of Methodological Quality

Characteristics of studies and risk of bias were extracted and assessed independently by four reviewers. After removing duplicate studies, a multistage screening process was performed to select studies meeting the inclusion criteria. In Stage 1, titles and abstracts of all identified records were screened by two authors (KK, KKE) based on the following criteria: age, healthy children and youth, empirical research or good-practice, school-based program, parental involvement in any form and output (categories were measured on a 3-point scale namely yes/no/unclear). Fifty per cent of all titles and abstracts were independently assessed by a second review author. All unclear studies were taken forward to the full-text screening at this stage. In Stage 2, full-text screening was performed where the four authors (KK, KKE, BZs, OÁ) independently screened the full texts following the criteria mentioned in Stage 1. In case of uncertainty, the other authors also checked the decision. All studies included in the review were assessed by two authors (KK, KKE) for methodological rigour. Due to the methodological diversity of the studies, Cochrane risk of bias tools [18,19] and the JBI critical appraisal checklist of the Joanna Briggs Institute (JBI) [20] were applied. Papers were evaluated according to the appropriate tool on a 6-point scale (Yes/Probably yes/No/Probably no/No information/Not applicable), a 5-point scale (Yes/Probably yes/No/Probably no/No information) or a 4-point scale (yes/no/unclear/not applicable) (see Section 3.1). Because of the heterogeneity of studies with respect to interventions, participants, measures and outcomes, no pooled effect sizes were calculated.

## 3. Results

Overall, the systematic searches resulted in 643 records. After filtering the title and abstract, 95 records were passed on for full-text filtering (Figure 1). All of these were examined for eligibility. A total of 17 articles met the criteria (see Table 1). The articles were published between 2008 and 2021, but most were published after 2010 (*N* = 16). As we found only two studies focusing on international differences, most of the studies present the research findings in the light of a national or regional study. Most studies were conducted in the United States (*N* = 7).

The studies used various methods: three studies were based on interviews, and three on cross-sectional studies. Three presented the results of a pilot-study, one introduced a noncontrolled trial, three introduced NRCTs, two presented RCTs, and one was a mixed-method study.

The spectrum of forms of parental involvement is very wide: leaflets, flyers, home instructions on healthy lifestyles (especially advice on physical activity and nutrition), school-based prevention programmes, meetings, consultations, advice, lectures with and led by experts and school-based programmes (sports games, competitions, gardening, cooking, etc.). Some of the studies examined the effectiveness of these programmes, and some included a parental dimension: they explored the difficulties and barriers to parental involvement by interviewing teachers, school administrators or school administrators of interscholastic sports (*N* = 4), while other, mainly qualitative studies examined parents’ opinions and attitudes towards these intervention programmes (*N* = 2). Three studies show the emergence of changes in learning and teaching caused by the pandemic. As part of this, involvement is shown in physical activity or even in the intervention programme, and it is introduced how this affected parents’ lack of choice to be involved in their children’s schoolwork since the spaces of the school, home and community merged.

### 3.1. Quality of Reporting

The results of the risk bias assessment and quality are introduced in Table 2. The identified two papers reporting RCTs [25,36] were assessed following the Cochrane ROB2 tool [18] to evaluate the quality of reporting. The ROB2 tool is a guideline specifically developed for improving the quality of reports on RCT. It is aimed to address the evaluation of nonpharmacologic treatments, such as behavioural interventions. Reports must be evaluated following the 21-item checklist on a 5-point scale (Yes/Probably yes/No/Probably no/No information). One introduced the Bienestar Health Program, a school-based diabetes prevention program [25]. However, this paper did not meet the ROB2 criteria and can be evaluated as having poor methodological quality, including some concerns concerning risk bias. The other paper introducing an RCT is also a healthy nutrition-related program introducing the Coordinated School Health Programs (CSHP) and had similar characteristics concerning bias and quality [36]. The main weaknesses were the lack of information on whether carers and people delivering the interventions were aware of participants’ assigned intervention during the trial, and the lack of information on whether outcome available for all, or nearly all, participants was randomised.

The three papers introducing NRCTs and the one presenting a noncontrolled trial were assessed on the Cochrane ROBINS-E tool [19], a tool developed to assess the risk of bias in the results of non-randomised studies comparing the health effects of interventions on a 6-point scale (Yes/Probably yes/No/Probably no/No information/Not applicable). The paper of Pippi et al. [30], focusing on Improving Umbrian Kids’ Healthy Lifestyle, that of Sormunen et al. [31], introducing the participatory action research of a Finnish health intervention program and that of Verjans-Janssen et al. [34], reporting the results of KEIGAAF intervention could be characterised with low risk of bias and fair quality. However, the noncontrolled trial of Barcelona et al. [21] lacked more components, showing a moderate risk bias and poor quality. The main weaknesses were the lack of information at the start of follow-up and start of intervention coinciding for most participants and the lack of information on participants excluded due to missing data on other variables needed for the analysis.

Cross-sectional studies were assessed on the JBI Checklist for Analytical Cross-Sectional Studies [20] where the content of the papers must be evaluated on a 4-point scale (yes/no/unclear/not applicable). All four studies could be regarded as papers with a low risk of bias. The study of Kehm et al. [28], reporting an intervention of school nutrition and physical activity, and that of Verhees et al. [33], introducing an intervention focusing on energy balance-related behaviours, can be assessed as having moderate quality. Meanwhile, the research of Williams and Mummery [35], focusing on the PRECEDE-PROCEED model to create healthy school environments and that of Xia et al. [34], discussing children’s sports participation could be evaluated with high quality. In some cases, objective, standard criteria used for measurement of the condition, confounding factors and strategies to deal with confounding factors were missing.

The four interviews and the qualitative pilot study were assessed on the JBI Checklist for Qualitative Studies [20], where the content of the papers must be evaluated on a 4-point scale (yes/no/unclear/not applicable). All of them could be evaluated as having a low risk of bias and high-quality methodology. In some cases, the statement locating the researcher culturally or theoretically or information on the influence of the researcher on the research were missing.

### 3.2. Target Populations in Children and Adolescents by Age and Social Background

The sociodemographic characteristics of the papers are introduced in Table 3. When analysing the sociodemographic characteristics of the target population, the most straightforward issue was the definition of age, with all but one study specifically defining the target age group of the participants in the programme or research. Most studies focused on children, 7 of them mainly on primary school children up to 11 years [23,24,25,30,31,32,34], and one study on those up to 12 years [36]. However, they were included in this group because the educational systems of the country and the target population of the programme tended to be in the lower age group. Four studies focused on upper primary and secondary school pupils [28,35,37], and one [27] focused on high school administrators involved in interscholastic sport. Three studies mixed the two age groups [21,26,33], one of which included results from interviews with primary school principals [22]. One study did not include the age of the children [26], as the qualitative research focused on parents and included parents with school-age children.

The results concerning socio-cultural and ethnic background were less clear. In most studies, the social background of the respondents or their participating children was not clearly defined [21,24,26,35]. Three studies explicitly targeted students of low social status [25,34,36], and one intervention involved middle-class Italian students [30]. Six studies involved subjects from mixed social backgrounds, and most of these examined differences in physical activity, nutrition and various health indicators across groups of students of different SES [22,28,31,32,33,37].

Looking at the ethnic background, we see a similar trend for SES. In the interview study carried out with administrators [27], this question was not relevant or clear. In seven studies, the ethnic background of the participants could not be identified [21,22,23,24,27,31,34,35], so a total of 9 studies were included in this category. Two studies dealt with Western students [30,32], one with Latinos [36] and one with Asian students (including one US immigrant) [29,33]. Three studies examined students from different ethnic and racial backgrounds (including one study of US-only minorities) [26,28,33], and one study selected children and parents not explicitly from an ethnic background but from Middle America, a specific geographic and cultural region in the US [25].

### 3.3. Methodological Diversity

The methodological characteristics of the papers are shown in Table 4. Concerning the quantitative research designs, one paper reported the results of a pilot study [29], and four were based on a cross-sectional study [28,33,35,37]. Concerning the qualitative studies, four studies used semi-structured interviews [22,23,26,32]. One paper was based on a mixed-methods study [27]. Concerning the trials, two papers reported the results of randomised controlled trials [25,36], three papers introduced non-randomised controlled trials [30,31,34], while one paper reported the results of a noncontrolled trial (pre–post-test) [21]. The methodological background is unclear in the case of one article [24].

### 3.4. Evidence of Effect on Health Behaviour among Children

In the next part of our analysis, we examined the impact of interventions that included physical activity or a sports programme on different dimensions of health behaviours, and the impact of parental involvement on an indicator related to a health behaviour or health indicator rather than an intervention, in an empirical analysis (Table 5). Eight studies did not include a specific intervention or did not focus on children but their parents [25,26,29,31,32], participating school staff [22,23] or interschool sports organisers [27]. In this case, participants were asked to report the perceived level and way of parental involvement in diverse contexts. For parents, they investigated parental involvement during the pandemic in supporting children’s education and, as part of this, in the delivery of school sports activities [29], two measured parents’ views and changes in the extent of their involvement and their own health behaviours [31,32], and two introduced the opportunities and barriers that prevented participation in the programme [25,26]. Of these, one study showed a positive effect of parental involvement on physical activity, child healthy lifestyle development, and psychological and social well-being [29], and the others [30] did not show any positive effect [22,23,25,26,27,31,32]. Most studies (overall 14) measured physical activity and performance in different forms (accelerometers, anthropometric and fitness tests, photovoice, questionnaires, interviews), either alone or in combination with other health behaviours and health indicators. Four studies found a positive effect on children’s physical activity [21,30,37], one of them from the parents’ perspective [29], and four studies found no clear effect [33,34,35,36]. The other important issue, nutrition, was found in one study to have a positive effect of the intervention [33]. In five studies, parents were also school staff members from the perspective of nutrition prevention programmes [22,23,25,26,32], with no analysis of changes in children’s eating habits. One study found a positive effect of parental involvement in school nutrition and physical activity policies and practices [28]. Three studies measured BMI change, two of which were not measured as one focused on parents [25], and one was unclear concerning the effect [36]. One study investigated BI; again, no clear evidence was found on whether there was an effect and in what direction [24], and as there was no measurement, the experience was summarised in the study.

### 3.5. Types of Parental Involvement

Papers introducing various types of parental involvement are introduced in Table 6. Based on the themes of the studies, two main strands can be distinguished concerning parental involvement: one group of studies includes those that identify forms of parental involvement (to the extent that they are presented), and the other group of studies mainly examines forms of parental involvement, their experiences or even barriers from the perspective of the participating school staff, or asked parents for their opinions and experiences of different school health programmes. The latter group included four studies exploring the forms, effects and opinions of parental involvement [27,29,32,37] and two studies on barriers and difficulties of parental involvement [22,26]. Regarding the content of the programmes, two were mainly educational programmes [30,34] or based on child-parent activities [25,33], and three were multi-component programmes, including training, meetings, workshops, information, newsletters and activities with the child inside or outside school [23,24,36]. One study reported a programme that primarily involved the distribution of information materials to parents [21]. One study also reported a specific dimension, the involvement of parents in school decision making for the planning and organisation of various health and prevention-related programmes. One study could not identify any kind of parental involvement [35].

## 4. Discussion

In our study, we searched for papers using school-based physical activity programmes with parental involvement. We aimed to identify studies with school sports or physical activity programmes that contribute to the development of students’ health awareness, health indicators (e.g., BMI, any dimension of health), or academic achievement, including some form of parental involvement. The search identified 17 studies that met the criteria. These studies describe interventions or programmes that primarily contribute to the development of two key health behaviours for obesity prevention: physical activity and conscious eating. Although our search covered any dimension of health, we did not find any studies that included smoking, alcohol or drug prevention or sexuality education, even if we defined the age of participants as up to 18 years. Only one study found behaviour change related to alcohol use and risk behaviours at the end of the programme, but this was also related to parents [31].

The current systematic review is an update to the review by Van Lippevelde et al. [13] in part. Our results confirm the main conclusion of their systematic review that there are very few good-quality interventions that address the role of parental involvement, and its impact is not clear, with only one study that clearly demonstrated a positive impact of the programme on participants’ physical activity and healthy lifestyle. The number of explicit follow-up studies is quite low, as only two of the original articles presenting the research results were based on an RCT, and three were original articles focusing on the results of NRCTs. This did not allow us to evaluate the effectiveness of intervention programmes in general. The involved original articles lack such data, making objective evaluation impossible. The presentation of methodological characteristics should be a decisive criterion for evaluating such programmes. In most of the studies analysed, the impact of the programme or parental involvement is unclear, with most of the positive effects being confirmed for physical activity. In two cases, we found studies, and systematic reviews, in which we read about positive or neutral effects. However, we did not find any literature where a negative effect was detected.

Parental involvement has a strong theoretical basis in education and psychology. The family functions as a system; thus, the subsystems known as family members have a significant and reciprocal impact on each other. According to the guidelines of systems theory, the system itself is qualitatively different, more than the sum of its parts, acting as a whole [20]. Therefore, changes in the child’s behaviour affect the parents’ behaviour and vice versa. However, the change in the child’s behaviour can be most effective and lasting when the parents’ attitudes and behaviour change in the right direction, consistent with the child’s attitudes and behaviour. Therefore, when designing an intervention, it is essential to consider the role of the family and parents to achieve the goal effectively.

The forms of parental involvement are extremely varied, including leaflets, home packs, pandemic “forced” sports organisations, parent meetings, programmes, courses, and school programmes with diverse children over several months (cooking together, gardening, playing sports together, etc.), which is why it is difficult to measure their effectiveness and impact. It should be noted that active participation in children’s activities and tasks tends to have a stronger impact on parents’ attitudes and behaviour because of the nature of the activities and the active participation itself. However, a weakness is the lack of active participation in some studies and programmes, which require passive participation from parents, e.g., reading brochures on a subject. Generally speaking, multi-component programmes that include activities with children in an organised way within the school, under the supervision and support of professionals, seem to be more successful. As Cook and Hayden [38] detailed in their work, parental information alone is not enough. However, the extent to which parents are involved in school life varies considerably from one social background to another [39] and is particularly marked for low SES and immigrant parents, for whom fear of expulsion from the country plays the most important role as a barrier to closer cooperation with any institution, including schools. It can be assumed that the limited effectiveness of the parenting component may also result in a lack of parental involvement. If passive participation is compared to active participation, e.g., doing different activities with the child, it can be assumed that the nature of the tasks is such that they are more effective and involve affective (e.g., the pleasure of the activity, the bond between parents and child) and cognitive components (e.g., presentations, brochures).

## 5. Conclusions

Parental involvement in school-based health programs can be a supportive factor in children’s positive and conscious formation of health behaviour [40]. However, the number of prevention and intervention programs and research focusing on this aspect is limited. Additionally, they take only a few aspects into regard. We could see only physical activity and dietary behaviour among the factors investigated, while health-risk behaviours were missing from such programs. However, it is well known that the high prevalence of health-risk behaviours is a persistent problem which usually appears in diverse prevention and intervention problems, but we have to note that they usually do not include parental involvement. Therefore, highlighting and incorporating parental involvement in such programs can significantly increase the efficacy of such programs. Increasing the use of passive (e.g., leaflets, flyers) and active components (e.g., meetings, consultations, advice, lectures) of parental involvement can significantly increase the efficacy of the prevention and intervention programs and the efficacy of the actors of the programs (including children, parents and professionals).

Another significant element that must be emphasised is the methodological quality of the papers. Although the risk bias was usually low or moderate in the case of the papers involved in the current systematic review, we have to highlight some problems. These were the lack of detailed information concerning the circumstances of the prevention and intervention programs, participants and experts involved in programs, research and assessment, or the objective and standard criteria used for measurement that complicated the interpretation of the results and their implementation in practice. Therefore, self-assessment and screening tools should be suggested for researchers to check the accuracy of their research, which can significantly improve the efficacy and validity of their research. They can support the researcher with a better reflection of the investigation and results and provide better quality evidence for professionals or decision makers from a long-term practical perspective.

## Figures and Tables

**Table 1:** Papers included in the systematic review.

Article Number	Author(s)	Date	Topic	Prevention or Intervention Program(s)	Location
[21]	Barcelona et al.	2021	healthy lifestyle	D-SHINES	USA
[22]	Clarke et al.	2017	obesity prevention	-	UK
[23]	Day et al.	2019	healthy lifestyle	Phunky foods	United Kingdom (England)
[24]	Evans et al.	2008	healthy lifestyle	CSHP	United Kingdom (England)
[25]	Garcia-Dominic et al.	2010	diabetes prevention	Bee star	USA
[26]	Ickes et al.	2016	healthy lifestyle	Challenge Club	USA
[27]	Johnson et al.	2019	sport	-	USA
[28]	Kehm et al.	2015	healthy lifestyle	-	USA
[29]	Kong et al.	2021	sport	-	USA
[30]	Pippi et al.	2020	sport	Improving Umbrian kids’ healthy lifestyle	Italy
[31]	Sormunen et al.	2013	health education	PAR project	Finland
[32]	Van Lippevelde et al.	2011	energy balance	ENERGY	Belgium, Norway, Hungary, Spain
[33]	Verhees et al.	2020	healthy lifestyle	Challenge Me	The Netherlands
[34]	Verjans-Janssen et al.	2018	healthy lifestyle	KEIGAFF	The Netherlands
[35]	Williams & Mummery	2015	obesity prevention	CHASE	Australia
[36]	Wright et al.	2012	healthy lifestyle	CSHP	USA
[37]	Xia et al.	2020	sport	-	China

**Table 2:** Quality assessment of the studies involved.

Authors	Study Design	Tool for Assessment	Risk of Bias	Quality
Garcia-Dominic et al. [25]	RCT	Cochrane ROB2 Tool	Some concerns	Poor
Wright et al. [36]	RCT	Cochrane ROB2 Tool	Some concerns	Poor
Pippi et al. [30]	NRCT	Cochrane ROBINS-E	Low	Fair
Sormunen et al. [31]	NRCT	Cochrane ROBINS-E	Low	Fair
Verjans-Janssen et al. [34]	NRCT	Cochrane ROBINS-E	Low	Fair
Barcelona et al. [21]	Noncontrolled trial (pre–post-test)	Cochrane ROBINS-E	Moderate	Poor
Kehm et al. [28]	Cross-sectional	JBI	Low	Moderate
Verhees et al. [33]	Cross-sectional	JBI	Low	Moderate
Williams & Mummery [35]	Cross-sectional	JBI	Low	High
Xia et al. [37]	Cross-sectional	JBI	Low	High
Clarke et al. [22]	Interviews	JBI	Low	Moderate
Day et al. [23]	Interviews	JBI	Low	High
Ickes et al. [26]	Interviews	JBI	Low	High
Van Lippevelde et al. [32]	Interviews	JBI	Low	High
Kong et al. [29]	Pilot-study (Qualitative)	JBI	Low	High
Johnson et al. [27]	Mixed methods	-	-	-
Evans et al. [24]	unclear	-	-	-

**Table 3:** Studies categorised according to the social-demographic background variables.

Social-Demographic Background	Studies (Article Numbers)
***Age***	
Children/elementary school pupils: 6–11 years	[23,24,25,30,31,32] (10–12 years), [36] (8–12 years)
Adolescence/upper primary or secondary school students: 12–18 years	[27] (interscholastic sports administrators, high school), [28,35,37]
Children and adolescence	[21,22] (with primary school principals), [26,33]
Unknown	[29]
***SES***	
Low SES	[25,34,36]
Middle	[30]
Mixed	[22,28,31,32,33,37]
Unknown/not relevant	[21,23,24,26,27] (interscholastic sport administrators), [29,35]
***Race/nationality***	
Western	[30,32]
Latino	[36]
Asian	[29] (US immigrants), [37]
Mixed	[26] (ethnic minority), [28,33]
other	[25] (Middle America)
Unknown/not relevant	[21,22,23,27] (interscholastic sport administrators), [31,34,35]

**Table 4:** Studies categorised by methodological quality.

Methodological Quality	Studies (Article Numbers)
Pilot study	[29]
Noncontrolled trial (pre–post-test)	[21]
NRCT	[30,31,34]
RCT	[25,36]
Mixed methods	[27]
Interviews	[22,23,26,32]
Cross-sectional	[28,33,35,37]
unclear	[24]

**Table 5:** Outcome effects of the intervention or parental involvement according to the type of outcomes.

Type of Outcomes	Outcome Effects of the Intervention or Parental Involvement (Article Numbers)		
	Positive	None or No Evaluation	Unclear
Anthropometric, Physical Performance, Activity	[21,29] (parents examined), [30,37]	[22] (school administrators examined), [23] (school staff involved in the programme examined), [25] (parents examined), [26] (parents examined), [27] (administrators examined), [32] (parents were surveyed),	[33,34,35,36]
School Nutrition And Physical Activity Policies And Practices	[28]		
BMI	[34]	[25] (parents examined)	[36]
BI			[24] (not measured)
Nutrition	[33]	[22] (school administrators examined), [23] (school staff participating in the programme examined), [25] (parents examined), [26] (parents examined), [32] parents examined	[36]
Lifestyle, Psychological And Social Well-Being	[29] (parents were examined), [30]		
Complex Health-Related Awareness-Raising		[31] (parents examined)	

**Table 6:** Studies categorised by the types of parental involvement and the topic of the study.

Types of Parental Involvement/Topic of the Study	Studies (Article Numbers)
Leaflets, newsletters, flyers, home packages	[21]
Training programmes: handshake, training, discussion, advice, consultation, workshop	[30,34]
Activities with your child (in or out of school, but organised by the school)	[25,33]
Involving parents at school decision-making level	[28]
Multi-component	[23,24,36]
Measuring the forms and impact of parental involvement and parents’ views	[27,29,32,37]
Exploring barriers to parental involvement	[22,26]
Unknown	[35]

## Data Availability

Data are available only on request due to ethical restrictions.
