# Correction: Kovács et al. The Effects and Types of Parental Involvement in School-Based Sport and Health Programs Still Represent a Knowledge Gap: A Systematic Review. *Int. J. Environ. Res. Public Health* 2022, *19*, 12859

**DOI:** 10.3390/ijerph20095677

**Published:** 2023-04-28

**Authors:** Klára Kovács, Karolina Eszter Kovács, Katinka Bacskai, Zsolt Békési, Ádám József Oláh, Gabriella Pusztai

**Affiliations:** 1 MTA-DE-Parent-Teacher Cooperation Research Group, Institute of Educational Sciences and Cultural Management, University of Debrecen, 4032 Debrecen, Hungary; 2 MTA-DE-Parent-Teacher Cooperation Research Group, Institute of Psychology, University of Debrecen, 4032 Debrecen, Hungary; 3 MTA-DE-Parent-Teacher Cooperation Research Group, Institute of Sports Science, University of Debrecen, 4032 Debrecen, Hungary

## Additional Affiliation

In the original article [1], there was an error regarding the affiliation for **Ádám József Oláh**. In his case, his affiliation **No. 2 (MTA-DE-Parent-Teacher Cooperation Research Group, Institute of Psychology, University of Debrecen, 4032 Debrecen, Hungary)** should be changed to **No. 3 (MTA-DE-Parent-Teacher Cooperation Research Group, Institute of Sports Science, University of Debrecen, 4032 Debrecen, Hungary)**.

## Reference List Correction

The authors would like to make corrections to the reference citations in the original article [1]. The order of some reference citations has been adjusted correspondingly. The corrected version of this literature review addresses errors in the numbering of citations that occurred in the previous version.

The authors apologize for any inconvenience caused and state that the scientific conclusions are unaffected. The original publication has also been updated.
